# A literature review addressing midwakh and e-cigarette use in the Gulf region

**DOI:** 10.1186/s42506-023-00146-4

**Published:** 2023-12-19

**Authors:** Sarah Dalibalta, Zinb Makhlouf, Layal Rabah, Fatin Samara, Yehya Elsayed

**Affiliations:** 1 Department of Biology, Chemistry & Environmental Sciences, American University of Sharjah, Sharjah, UAE; 2 Advanced Research and Development, Fiber Media at Donaldson, Donaldson, MN USA

**Keywords:** Smoking, Dokha Midwakh e-cigarettes, Alternative tobacco products, Arabian Gulf countries, Health impact, Chemical composition

## Abstract

A notable decrease in conventional cigarette smoking has been witnessed on a global scale. However, this decrease has been accompanied by an equally striking global increase in the consumption of alternative tobacco products (ATPs), namely e-cigarettes and midwakh in the Arabian Gulf region. A literature review was used to outline the chemical composition of these two ATPs and review their impacts on health. The study was conducted using databases like PubMed, Google Scholar, MDPI, and WorldCat. The literature search included terms such as “e-cigarettes,” “midwakh,” “dokha,” “heath impacts,” “psychological effects,” “social influences,” and “cigarette smoking” with emphasis on literature from the Arabian Gulf region. Data shows that midwakh contains markedly high levels of tar, nicotine, and various compounds of notable effects on the human body. Similarly, it was found that e-cigarettes contain non-negligible amounts of nicotine and other chemical compounds that may not have been extensively investigated. Alarming reports of system-specific effects brought about by midwakh, and e-cigarette consumption, have been reported, although further research is needed to deduce the mechanism. We also discussed some of the social and psychological factors leading to their consumption within this population. Hence, this review raises questions around the safety of these two types of ATPs and encourages comprehensive studies globally and regionally.

## Introduction

A recent decrease in the consumption of conventional cigarettes has been witnessed globally, with a variety of national and international studies confirming this trend [1–5]. Between 2009 and 2017, global cigarette smoking prevalence declined by 7.7% in men and 15.2% in women [6]. This can be explained by the control measures taken by countries against tobacco use including increasing awareness programs on its dangers and banning tobacco usage in public places [6]. Another potential explanation for this significant reduction is the reported correlation between higher cigarette pricing and smoking cessation [7]. At a first glance, this decrease may seem like a positive skew; however, the decrease in conventional cigarette smoking is coupled with an exponential increase in alternative tobacco products (ATPs) [8].

ATPs include midwakh (dokha), electronic nicotine delivery systems such as electronic cigarettes (e-cigarettes/vapes), smokeless tobacco (chewing tobacco), pipes, hookah (water pipes), and recently marijuana [9–11]. Despite the extensive list of ATPs, we will describe two products that prevail in the Gulf Cooperation Council (GCC) countries, namely e-cigarettes, which have seen a global rise [12–15], and midwakh, which is growing in popularity locally [12–15]. In the United Arab Emirates (UAE), 18.5% of the surveyed population disclosed trying midwakh, and 9% are current midwakh smokers [13]. According to Al Sharbatti et al., within 54.4% of midwakh smokers in Ajman, 48.9% of the participants were male adults between the ages of 26–35 [15]. Another study revealed that most participants were e-cigarette smokers of 40 years of age or less [16]. Also, out of 15.1% of students from three UAE universities who are current smokers, 44% are e-cigarette and midwakh consumers [17]. In Saudi Arabia, 7.9% of male college students have smoked midwakh, and 3.8% are current midwakh smokers [18]. Remarkably, 12.2% of medical students in Saudi Arabia are current e-cigarette smokers [19]. In Qatar, 14% of university students use e-cigarettes, and 4.7% of surveyed Qatari smokers use midwakh [20, 21]. In Kuwait, a study showed 26.4% of high school students are current e-cigarette users [22].

Although deemed as a growing public crisis, little is known about the health effects of ATPs [8, 23, 24]. Additionally, many e-cigarette consumers are not using ATPs to cesate smoking [25]. According to the World Health Organization (WHO), adolescents and children who use electronic nicotine delivery systems are more than twice as likely to use conventional cigarettes [11]. Compelling evidence implies that e-cigarette consumption may lead to eventual conventional cigarette smoking among youth [26]. This review aims to describe the chemical composition of e-cigarettes and midwakh, along with their health effects as well as some of their social and psychological impacts, especially within the Gulf region.

## Methods

A literature review was conducted using multiple databases including WorldCat, PubMed, MDPI, and Google Scholar to gather relevant studies essential for this review. The search was conducted between March 2022 and March 2023 and focused mainly on studies underlying the effects of e-cigarettes and midwakh (dokha) on health within the Arabian Gulf regions. Keyword search was used to assist with the database search which included the following: “e-cigarettes,” “midwakh,” “dokha,” “Arabian Gulf,” “heath impacts,” “psychological effects,” “social influences,” and “cigarette smoking.” After extensive research, the current review incorporated 87 research and peer-reviewed papers focusing on the impact of e-cigarette and midwakh within the Gulf region.

## Results

### The chemical composition of ATPs

#### The chemical composition of dokha

Dokha is a blend of tobacco leaves with an array of spices, herbs, barks, dried fruits, or dried flowers and has gained its name from the pipe used to smoke the blend, the “midwakh” [12]. The array of available flavors misrepresents midwakh as a healthier cigarette counterpart among young people population. In fact, a few studies have investigated the role of toxicants and heavy metals in midwakh smoking-related diseases [27, 28]. Yet, studies that did analyze the chemical composition of midwakh report non-negligible findings [28]. As mentioned, midwakh is composed of fine dried green tobacco leaves collected without major processing. They retain high amounts of carbon monoxide, nicotine, and tar [29].

Additionally, midwakh is often smoked without a filter and rarely with a resin filter causing toxicants such as tar, carbon monoxide, hydrogen cyanide (HCN), nitrosamines, volatile organic compounds, and toxic heavy metals to immediately enter the lungs [29–31]. Tar, the sticky, aerosol residue of tobacco combustion, contains most of the toxic, carcinogenic, and mutagenic agents in tobacco [29]. Midwakh (dokha) has strikingly higher nicotine and tar levels than conventional cigarettes. A recent study reports that nicotine in different midwakh brands falls between 23.82 and 52.80 mg/g [29]. Furthermore, midwakh has 55.62% higher tar concentrations than cigarettes [29, 30]. CNS depressants and carcinogens such as acrylonitrile, benzene, and acrylamide have also been detected in midwakh [28, 29]. Moreover, concentrations of trace metals such as lead, cobalt, aluminum, nickel, copper, chromium, manganese, iron, potassium, calcium, zinc, magnesium, and strontium are markedly higher in midwakh than in cigarettes [27–29].

Alarmingly, metal content in midwakh is unregulated. A chemical analysis of midwakh smoke identified over 400 organic compounds including 22 irritants; 5 toxic compounds such as cycloheptatriene, 2-methylfuran, and m-xylene; and at least 3 carcinogens including benzene [28]. A midwakh tobacco brand was shown to have concentrations of aluminum, boron, cobalt, copper, lead, and zinc at 421.2 μg/g, 219.8 μg/g, 25.1 μg/g, 24.0 μg/g, 468.6 μg/g, and 342.7 μg/g, respectively [28]. These metals are in quantities equal or higher than in cigarettes. Interestingly, polycyclic aromatic hydrocarbons (PAHs), which are usually formed during incomplete combustion, were also detected in midwakh samples [31]. Two PAHs, naphthalene, and anthracene were found in trace amounts in raw dokha tobacco, while 12 PAHs were found in midwakh smoke at concentrations surpassing those detected in cigarettes [31].

#### The chemical composition of e-cigarettes

Formerly produced as a tobacco cessation facilitator, e-cigarettes are noncombustible electronic nicotine delivery systems containing flavoring agents [24, 25]. While e-cigarettes advanced from first-generation pods with disposable e-liquid cartridges to third-generation e-cigarettes with a refillable e-liquid tank and then to fourth-generation Joel brand, e-cigarette smoking essentially involves heating and aerosolizing an “e-liquid” made of nicotine, propylene glycol (PG), vegetable glycerine (VG), and flavor compounds using a battery-mediated device [32]. This resistance heating is done through a metallic coil, which is commonly Kanthal, comprised of iron, chromium, and aluminum or nichrome, a coil consisting of nickel and chromium [32]. Consequently, due to the thermal degradation of the e-liquid, e-cigarette gas and particle emissions consist of aerosolized PG, VG, flavors, nicotine, free radicals, and various carbonyls, and an array of hydroxycarbonyls were reported [32]. Carbonyl compounds including acetaldehyde and formaldehyde, which normally form after heating, have also been detected in e-cigarette vapor, in lower levels than cigarette smoke [33].

Unlike their conventional counterpart, no precise protocols have been established to test e-cigarettes, yet chemical analyses have been carried out, whereby 46 volatile and semi-volatile compounds were detected in e-liquid formulations and 55 compounds were found in e-cigarette aerosols [34]. A study investigating the presence of carbonyl compounds, volatile organic compounds (VOCs), tobacco-specific nitrosamines (TSNAs), and metals revealed that all tested e-cigarette samples contained three carbonyls with reported toxic properties: formaldehyde, acetaldehyde, and acrolein [35]. Almost all samples contained the volatile organic compounds toluene and m, p-xylene, and all generated vapors containing nickel, and lead, with some vapor samples containing traces of the carcinogenic nitrosamines: N′-nitrosonornicotine (NNN) and 4-(methylonitrosoamino)-1-(3-pirydyl)-l-butanone (NNK) [35].

An elemental analysis of thirty-six inorganic chemical elements revealed that e-cigarette aerosols include a variety of elements encompassing heavy metals at concentrations significantly higher than in conventional cigarettes [36]. Further analysis showed that e-cigarette fluid and aerosol contain nickel, chromium, copper, zinc, silver, and lead [37]. In order to compare metal concentrations in e-liquids from the refilling dispenser, e-liquids in the tank, and the inhaled aerosol, an analysis was carried out using samples from the devices of daily e-cigarette users. It revealed that concentrations of most metals were significantly higher in samples collected from tanks and the aerosols to be inhaled by the consumer than those from the refilling dispenser [37]. Concentrations of chromium, copper, nickel, lead, and zinc were also more than 25 times higher than in the dispenser samples, and the concentrations of aluminum, cadmium, and antimony were between 2.30 and 4.65 times higher in the tank and between 1.60 and 3.58 times higher in the aerosol in comparison with the dispenser samples [37].

Flavoring compounds contain diacetyl and various aldehydes including benzaldehyde, vanillin, ethyl vanillin, and cinnamaldehyde noting that many of the employed flavorants have been deemed as safe food additives. However, their consumption through inhalation has not been designated as safe by the Federal Food, Drug, and Cosmetic Act (FFDCA). Aldehydes are reactive and can produce adducts by forming covalent bonds with nucleic acids, cellular proteins, and other biomolecules [38]. Interestingly, detected concentrations of aldehydes vary greatly among commercial e-cigarettes liquids and can be as high as 34% as in the case of cinnamaldehyde in cinnamon flavored e-liquids [39]. Acetoin, diacetyl, and its structural analogue 2,3-pentanedione are used in various food flavorings such as butter, caramel, and strawberry, but they are also commonly employed in e-cigarette flavors with strong appeal such as cupcake, cotton candy, and Blue Water Punch [40]. In their study of 51 e-cigarette flavors, Allen et al. disclose that at least one of these three compounds was detected in 47 of different flavor samples, and that diacetyl, a chemical compound correlated with various severe respiratory pathologies in microwave popcorn-processing plant workers, was observed in concentrations above the laboratory limit in 39 of these flavors [40] (Table 1).

**Table 1:** The chemical composition of dokha [28–31] and e-cigarettes [32–40]

Reported findings on chemical composition	*Midwakh*	*E-cigarettes*
**Chemicals**	Tar Nicotine Carbon monoxide Hydrogen cyanide (HCN) Nitrosamines Cycloheptatriene 2-Methylfuran M-xylene Benzene	Nicotine Flavoring agents Propylene glycol (PG) Vegetable glycerine (VG) Hydroxycarbonyls Acetaldehyde Formaldehyde Acrolein N′-nitrosonornicotine (NNN) 4-(Methylonitrosoamino)-1-(3-pirydyl)-l-butanone (NNK) Benzaldehyde Vanillin Ethyl vanillin Cinnamaldehyde
**Trace metals**	Acrylonitrile Acrylamide CNS depressants Lead Cobalt Aluminum Nickel Copper Chromium Manganese Iron Potassium Calcium Zinc Magnesium Strontium	Iron Chromium Aluminum Nichrome Copper Zinc Silver Lead

### The health effects of ATPs

#### The health effects of dokha

Unlike the effects of tobacco smoking on health that have been very well documented, the effects of ATP consumption are poorly studied. Various authors report a scarcity in studies investigating the health effects of midwakh consumption [41–43]. One report shows that midwakh smoking has serious effects on blood pressure like other forms of smoking [12]. Notably, midwakh contains higher concentrations of nicotine and tar than other tobacco products [43, 44]. Since nicotine levels in midwakh (23.83–52.80 mg/g) are much higher than those in cigarettes (0.5–19.5 mg/g), they are expected to impose harmful health effects [29]. The alkaloid, nicotine, can permeate the blood-brain barrier; bind to nicotinic receptors in the central nervous system, promote adrenaline release; and, consequently, stimulate cardiac contractility and constrict blood vessels [44]. In chronic use, excessive sympathetic stimulation leads to continuous elevation of heart rate and cardiac output, causing flow turbulence and potentially damage to blood vessel lining [44].

A study investigating the effects of midwakh smoking on male UAE medical students revealed a significant mean increase in systolic blood pressure of 12 ± 1 mmHg, 20 ± 2 bpm in heart rate, and a nonsignificant mean decrease in diastolic blood pressures of 1 ± 1 mmHg [43]. In other words, midwakh smoking appears to increase heart rate and systolic pressure significantly. Shaikh and colleagues report that immediately after smoking midwakh, there was an observed increase of 4 ± 1 breaths/min in respiratory rate by (2 ± 2 breaths/min) [43]. This is higher than the increase (in respiration rate) brought about by waterpipe smoking. Given that midwakh smokers smoke dokha an average of 12 times per day, they are exposed to markedly high levels of nicotine and tar, increasing their risk of lung cancer [44, 45]. While not considered a carcinogen, nicotine has been associated with bronchial epithelial cell apoptosis [46]. Midwakh analyses revealed high toxin levels and five central nervous system depressants in its smoke [28]. A growing number of seizure cases following midwakh consumption have also been reported in the literature. For example, after a sustained seizure following midwakh consumption for the first time, a 17-year-old male was rushed into an emergency unit and presented with confusion, tachycardia, tachypnea, and a blood pressure of 180/100 mm Hg [42].

Other similar cases were reported by Alsaadi and colleagues [47]. Remarkably, seven male adolescents, who were otherwise healthy, were admitted to hospital for new onset tonic-clonic seizures after a few minutes of smoking midwakh [47]. While further research is required to pinpoint the mechanisms behind midwakh-induced seizures, the high nicotine content in addition to the potential effects of the additives may have brought these about [47]. Ultimately, these few studies point to a substantial psychological and health risks that should be properly evaluated.

#### The health effects of e-cigarettes

E-cigarettes have been regarded as the less harmful alternative to cigarettes [48]. Since conventional cigarette smoking is typically completed in 8–10 puffs over a 5–8-min period, most e-cigarette smoking is intermittent throughout the day, leading to lower and more stable nicotine levels with no arterial spikes [49]. Some studies report that e-cigarette consumption poses low cardiovascular risk, at least when it comes to short-term use in healthy users [48, 49]. Yet, other studies investigating the longer cardiovascular repercussions of e-cigarette smoking are limited and controversial since novel compounds in e-cigarette vapor, such as flavorings and fragrances, are mostly untested, and their cardiovascular effects are unexamined [50, 51].

Interestingly, a meta-analysis including studies published in 2000-2017 reported negative effects of e-cigarettes on endothelial function, an increase in arterial stiffness, and a greater long-term risk for coronary events [51]. Despite the reported negative effects of e-cigarettes on heart rate, diastolic and systolic blood pressure, one study reported that switching from conventional smoking to e-cigarettes had positive effects on blood pressure regulation [52]. However, dual smokers of e-cigarettes and combustible cigarettes were 36% more likely to suffer from cardiovascular disease [52]. An inhalation toxicology analysis revealed an absence of oxidative stress and inflammation in mice exposed to e-vapor aerosols, while high urinary markers were detected in mice exposed to conventional cigarette smoke [53]. E-cigarette vapor aerosols produced smaller atherosclerotic plaques, affected systolic and diastolic cardiac function, and endothelial function in significantly less severity than conventional cigarettes [54].

A recent case report disclosed that an otherwise healthy patient without any previous cardiopulmonary comorbidities developed a sudden severe, acute cardiomyopathy with e-cigarette use [54]. Another study reports the association of daily e-cigarette use, but not former or occasional e-cigarette use, with higher risks of myocardial infarction in comparison with conventional cigarette use [55]. E-cigarettes also release various compounds displaying pulmonary toxicity such as volatile carbonyls, reactive oxygen species, furans, and metals [56]. A few comprehensive studies investigated the long-term effects of e-cigarettes compounds such as vaporized nicotine and its associated solvents, PG, and VG [57, 58]. E-cigarette aerosol inhalation has been reported to produce enhanced airway reactivity, airway obstruction, inflammation, and emphysema [58]. After exposure to e-cigarettes, airway irritation, mucus hypersecretion, inflammatory response, systemic changes, and altered respiratory function were observed [59, 60].

Moreover, e-cigarette exposure could lead to alteration in gene and protein expression, inhibition of ciliary beating, inhibition of cystic fibrosis transmembrane conductance regulator, and increased cytokine expression in bronchial epithelia [57]. Similarly, the nasal epithelia display inhibited ciliary beating and downregulation of immune genes, while the bronchi display increased stiffness and impaired vasoconstriction [57]. In fact, e-cigarette consumption is linked with aggravated pathology in asthma, cystic fibrosis, and chronic obstructive pulmonary disease patients [60]. The most imminent of all e-cigarettes pulmonary risks is the e-cigarette-associated lung injury (EVALI) [60]. For instance, a case report disclosed that a 35-year-old female electronic nicotine delivery system user presented to the emergency department with sudden-onset dyspnea [61]. Bronchoscopy revealed an extensive pattern of potential airway chemical injury, implying vesicular bronchial injury ensuing e-cigarette use [61]. Another case of a healthy 31-year-old male presenting with lung damage met the CDC definition for potential e-cigarette smoking-associated lung injury [62]. Interestingly, these health hazards are reported to be independent of nicotine. Madison and colleagues [57] report that independent of nicotine, ENDS-exposed mice infected with influenza demonstrated enhanced lung inflammation and tissue damage, and impaired alveolar macrophage physiology, which may be mediated through PG/VG solvents, which are currently deemed safe. Hence, future studies should investigate the direct effects of PG and VG used in e-cigarettes.

Moreover, 35 seizure cases ensuing e-cigarette consumption were reported to the FDA and poison control centers between 2010 and early 2019, and since reporting is voluntary, there is a possibility that even more cases exist [63, 64]. In fact, as of March 2021, more than 250 reports on e-cigarette-associated seizures showed that about 2/3 of the cases were presented in adolescents [64]. Yet, these reports provide limited information on the medical evaluation of each case. Since correlation does not necessarily imply causation, the FDA has requested elaborate assessment of e-cigarette use in patients presenting with e-cigarette-associated seizures to decipher whether nicotine is the mediator of these neurological symptoms [64].

Since nicotine is present at higher concentrations in conventional cigarettes and no other nicotine intoxication has been mentioned, other compounds present in e-cigarettes may be the culprits, especially since many of e-cigarette liquids can be mixed with seizure inducers such as caffeine or synthetic cannabinoids [65]. Aside from this increase in reported seizures, little is known about e-cigarette effects and neurotoxicity [66, 67]. A murine study reported that e-cigarette exposure for 7 days downregulated GLUT1 and GLUT3 expressions and hence glucose uptake. This induced glucose deprivation could enhance ischemic brain injury and/or stroke risk [68]. E-cigarette smoking could therefore be a promoting factor for stroke onset, deterioration of postischemic brain injury, and loss of blood-brain barrier integrity [68].

E-cigarette consumption seems to expose adolescents to lifelong alterations in neuronal signaling, which affects behaviors ranging from emotional regulation to addiction [69]. In fact, e-cigarette smoking has been associated with various drug use and mental problems such attention-deficit/hyperactivity disorder, posttraumatic stress disorder, gambling disorder, anxiety, low self-esteem, and impulsivity [69, 70]. Recent studies reveal a correlation between depression and e-cigarette consumption and an association between greater depressive symptoms at the age of 14 and faster e-cigarette escalation [71, 72]. Of note, e-cigarette consumption may affect people surrounding the user. The mainstream vapor exhaled by the user contains vaporized particles and droplets that can have significant health effects on the passive inhaler. It is worth mentioning that indoor e-cigarette exposure potentially exposes nonusers to nicotine only and not toxic tobacco-specific combustion products [73]. Other studies [75–79] reported other physical and mental health effects with cancer as the most serious [77, 79] (Table 2). Interestingly, an e-cigarette health warning could potentially encourage users to quit e-cigarette smoking and discourage smoking [73, 74]. Future efforts should be directed towards clearing the misconceptions around the assumed safety of e-cigarettes.

**Table 2:** Reported health effects of cigarettes in comparison to e-cigarettes and midwakh

Impact of smoking on health	*Cigarettes*	*E-cigarettes*	*Midwakh*
**Reported cardiovascular complications**	Myocardial infarction [49] Atherosclerosis [49] Hypertensive heart Disease [49] Hypertension [49] Hypercholesterolemia [74]	Increase in heart rate [58] Increase in systolic pressure [58] Increase in cardiac contractility [58] Constriction of blood vessels [58]	Increase in heart rate (20 ± 2 bpm) [43, 75] Increase in systolic pressure (12 ± 1 mmHg) [43, 75] Increase in diastolic pressure (1 ± 1 mmHg) [43] Increase in cardiac contractility [75] Constriction of blood vessels [75]
**Reported respiratory symptoms**	COPD [49] Chest tightness [76] Coughing [76] Chronic bronchitis [77] Pulmonary emphysema [77]	COPD [58] Chest tightness [78] Coughing [78] Wheezing [78] Bronchial inflammation [78] Interstitial lung diseases [58]	Increase in respiratory rate (4 ± 1 breath/min) [43, 75]
**Reported cancer risk**	Lung cancer [49] Mouth cancer [74] Esophageal cancer [74] Colon cancer [74] Pancreatic cancer [74] Breast cancer [74] Laryngeal cancer [74] Kidney cancer [74] Leukemia [74] Acute myeloid Leukemia [74] Liver cancer [77] Stomach cancer [77]	Lung cancer [79]	Lung cancer [75]
**Reported findings on other health impacts**	Type 2 diabetes [49] Dyslipidemia [77] Stroke [49] Reproductive disorders [49] Hypertensive renal Disease [49]	Gingival pain [78] Bleeding [78] Oral pain [78] Cracked/broken teeth [78] Inflammation [78] Gum disease [78] Depression [78]	

## Discussion

Midwakh use in the UAE accounts for 15% of the total tobacco users [15]. The use of midwakh has been gaining interest, mainly because it is cheaper and easy to use. According to Shemmari et al., smoking dokha is considered appealing especially by the younger age groups because it is less expensive than smoking cigarettes, with a week’s supply costing around US $3 versus US $21 for cigarettes. In addition, the lack of odor, the absence of lip staining, and the speed at which the effects are felt allow the smoker to practice rapid smoking in a discreet manner [80]. In some countries, water pipes (also known as shisha) and midwakh are more socially acceptable types of tobacco use, and most of the people who smoke midwakh, according to a study in UAE, do so because of peer pressure, stress relief, or simply for the experience [76].

The lack of barriers and ease in ability to obtain tobacco, and the financial standing of the nation, all have sway over the potential to create new smokers, especially in the young population [81]. Moreover, Elobaid et al. [82] revealed that stress was the main factor to smoking midwakh (dokha) among male students, especially during examinations. The UAE has established various policies to regulate the selling and consumption of midwakh, by penalizing smoking in private cars in the presence of a child under 12 in confined public areas, worship areas, educational establishments, and sports and health facilities. This law also forbids selling tobacco products to those under 18, selling sweets or candies that have similar appearances to tobacco products, and creating automatic vending machines or devices that distribute tobacco across the country, in addition to forbidding advertising tobacco products [83].

In addition, Cabral, [84]**.** discloses that factors like loneliness, stress, and depression were linked with smoking e-cigarettes among university students as a coping mechanism, especially during COVID 19. Park et al. [85] measured psychological factors like depression, anxiety, and hopelessness, among e-cigarette and cigarette smokers. Results revealed that both had high levels of psychological stress during every smoking session [85]. The influence of a family member or friend was also one of the main reasons associated with the use of e-cigarettes, specifically among adolescents [86]. In a study among young adults in UAE, 27.1% of the surveyed e-cigarettes users had at least one parent that smokes one of the ATPs, and 44.6% had a friend that smokes [86]. A total of 34.4% of students surveyed, among three public UAE universities, mentioned that different flavors of e-cigarettes encouraged them to smoke, while others reported that e-cigarettes seemed like a healthier option than traditional smoking [17]. The abundance of flavors is linked to satisfaction and addiction among young adults, possibly encouraging both e-cigarettes and midwakh smoking between adolescents [87].

## Conclusions

Theoretically, the ATPs purpose was to facilitate smoking cessation among the cigarette smoking population. In fact, its use has been predicted to encourage utilization of other substances. Yet, information on its chemical and physiological effects is alarming. *Compelling* evidence implies that e-cigarette consumption may lead to eventual conventional cigarette smoking with an alarming increase in e-cigarette consumption among youth. Of note, some studies reveal that ATPs were encouraged within social activity with peers. Smoking dokha and e-cigarettes, however, may be a coping mechanism underlying psychological stress. Depression was commonly found amid e-cigarette smokers and more likely to occur in e-cigarette smokers than nonsmokers, specifically among young adults. With social influences and emerging flavors being one of the main reasons behind smoking e-cigarettes and midwakh, there is a concerning growth in public health among adolescents. Despite many studies indicating the harmful effects of e-cigarettes and midwakh on physical and mental health, it is still a controversial topic that requires a lot more research and regulation.

## Data Availability

The datasets used and/or analyzed during the current study are available from the corresponding author on reasonable request.
